# A huge appendiceal mucocele

**DOI:** 10.11604/pamj.2022.43.123.33175

**Published:** 2022-11-04

**Authors:** Mohamed Al Amine El Mouden, Said Ait Laalim

**Affiliations:** 1 Department of General Surgery, University Hospital Center, Tangier, Morocco

**Keywords:** Mucocele, appendix, appendicectomy, surgery

## Image in medicine

Appendiceal mucoceles occur when there is an abnormal accumulation of mucin causing abnormal distention of the vermiform appendix due to various neoplastic or non-neoplastic causes. The prevalence at appendectomy is 0.2-0.3%. They affect the middle-aged individuals, considering the epidemiology of the mucinous neoplasms. Though carcinoid tumor is the most common primary appendiceal neoplasm in surgical pathology series, mucoceles due to neoplasms are the most common appendiceal tumors detected on imaging. We report the case of a 54-year-old patient with no pathological history, who arrived at the emergency department presenting an appendicular syndrome evolving for a week with fever and vomiting, without transit disorders or urinary signs. Physical examination found tenderness of the right iliac fossa. An ultrasound was performed urgently and showed an appearance of a superinfected appendicular mucocele. The patient was admitted to surgery room 2 hours later. Surgical exploration objectived the presence of a very long appendix measuring 15cm x 2cm with an unthickened ceaco appendicular junction. The rest of the peritoneal cavity was unremarkable. An appendicectomy was performed. The anatomopathological examination objectived a ruptured chronic appendicular mucocele low grade mucinous neoplasia (LAMN).

**Figure 1:**
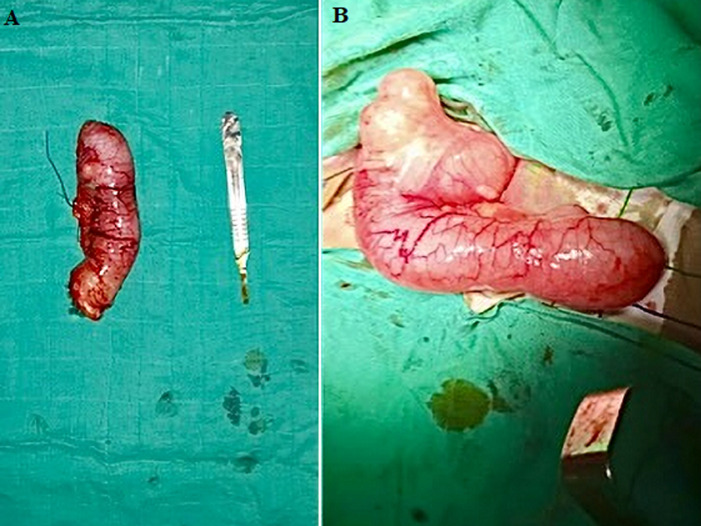
appendicular mucocele; (A) a large appenditis measuring 15cm x 2cm discovered intraoperatively which evokes an appendicular mucocele; (B) an appendectomy was performed on a normal-looking part of the cecum with associated resection of the mesoappendix

